# Toward a conceptual framework of economic security for health research: a scoping review

**DOI:** 10.3389/fpubh.2026.1887796

**Published:** 2026-10-26

**Authors:** Brian J. Stevenson, Amanda K. Falcón, Bo Kim, Taylor Hunt, Marc I. Rosen, Jack Tsai, Kendra E. Pugh, Mayumi O. Gianoli, James P. LePage

**Affiliations:** 1 VA Bedford Healthcare System, Bedford, MA, United States; 2 Department of Psychiatry, UMass Chan Medical School, Worcester, MA, United States; 3 VA Boston Healthcare System, Boston, MA, United States; 4 Department of Psychiatry and Harvard Medical School, Boston, MA, United States; 5 VA Connecticut Healthcare System, West Haven, CT, United States; 6 Department of Psychiatry, Yale University School of Medicine, New Haven, CT, United States; 7 National Center on Homelessness Among Veterans, United States Department of Veterans Affairs, Washington, DC, United States; 8 Department of Management, Policy, and Community Health, University of Texas Health Science Center at Houston, Houston, TX, United States; 9 Veteran Employment and Economic Security Research Consortium, VA North Texas Health Care System, Dallas, TX, United States; 10 Department of Psychiatry, University of Texas Southwestern Medical Center, Dallas, TX, United States

**Keywords:** economic insecurity, economic security, economic security outcomes, financial instability, health

## Abstract

**Background:**

Personal economic security, including stable income, employment, affordable housing, and protection against economic shocks, is fundamental to health. However, multiple and varied frameworks exist for defining or measuring economic security in health research and practice.

**Methods:**

We conducted a scoping review of the economic (in)security literature published 2015–2025. We searched PubMed and PsycINFO for articles that defined or measured economic (in)security, or that examined the impact of economic (in)security on health-related outcomes. From 881 sources, 53 articles were reviewed. Definitions and methods of operationalizing economic security were extracted and coded based on thematic dimensions, domains, and sub-domains.

**Results:**

There is wide variability in definitions and measurement of economic security. Economic security was defined multi-dimensionally across thematic domains, subjectivity and objectivity, and temporal dimensions (present versus future risk). Operationally, six domain areas of economic security were identified: income/finances, healthcare, employment, housing, food, and wealth/assets.

**Conclusions:**

This six-domain framework of economic security for health research provides a foundation for more consistent measurement, improved intervention design, and specification of pathways linking economic conditions to health outcomes. Further investigation is needed to examine the framework's utility in elucidating economic security's multi-dimensional nature and devising measurement strategies to advance the science and practice of improving economic security.

## 1 Introduction

Economic insecurity is identified as an international concern and priority. At a global level, the United Nations' (UN) Department of Economic and Social Affairs posits that increased economic insecurity among a country's citizens contributes to the destabilization of societies worldwide through increased polarization and distrust (1), while affecting the most vulnerable citizens including younger and older adults, those with lower education, and those from racial and ethnic minorities (2).

National and pan-national efforts have increasingly focused on improving the economic security of citizens at both national and individual levels. The European Union (EU) has drawn increased attention to economic insecurity by exploring and calling for better coordination between its members in improving economic safety nets for its citizens, including recommendations for harmonization of minimum incomes and protections across member nations. Within the United States (US), the Department of Labor, the Federal Reserve System, and the Census Bureau provide publicly available statistics on the economic security of workers and citizens, while the Department of Veterans Affairs (VA) Office of Research and Development has identified economic insecurity as a priority research focus.

While economic factors have traditionally been seen as outside the realm of healthcare, a growing body of research and public health programs now position them as integral to achieving sustainable health (3–5). Relationships between indicators of economic insecurity (e.g., material deprivation, poverty, etc.) and innumerable health outcomes have been found across a variety of mental health (6) and physical health (7, 8) outcomes. Starkly, emerging evidence suggests that poorer county-level economic security is associated with higher rates of “deaths of despair” (e.g., overdose, suicide) (9, 10).

To assess economic insecurity, researchers and policymakers often rely on factors such as poverty or other indicators of socio-economic status (e.g., household income). The term “poverty” is frequently tied to a combination of income and family composition where income fails to meet basic needs (11). Though these terms are easily understood on the surface, they may serve as a poor proxy for economic insecurity. For example, poverty definitions may vary between regions or countries (12), or even within government agencies (11), limiting generalizability. Using a broad definition of poverty may not capture pressures or sources of pressure caused by economic factors that may lead to behavior changes (13). A focus on poverty level indicators also may exclude individuals from receiving needed resources that are not captured by stringent poverty limit thresholds (14, 15). Additionally, the term “poverty” has seen an expansion in scope to include a range of factors including access to decent work, social protection, and human rights (16), resulting in possibly reduced precision in classification.

Using poverty as a proxy for economic insecurity also fails to encompass central components of economic insecurity: anxiety and stress (17–19). In a United Nations' Department of Economic and Social Affairs report (18), the UN recognized that though no explicit definition for economic security has been developed, economic insecurity encompasses worrying about or fearing economic conditions and hazards. These hazards have two components, the magnitude of the future harm and the probability of it occurring. In situations where the economic outlook is bleak, social relationships and emotional wellbeing will suffer. One recent review of the literature (20) summarized the general state of defining economic insecurity as an exposure to current or future financial distress, future economic loss, and difficulties mitigating consequences of future economic events.

Economic insecurity has been shown to have a broad impact on health at an individual level, primarily through the vector of insecurity related stress. Stress associated with economic insecurity has been shown to be positively associated with depression (21, 22), anxiety (23), negative partner relationships (24), drug use (25), and broad psychological distress (26). Economic insecurity also has downstream impacts on health by increasing the likelihood of intimate sexual violence (27), remaining in violent relationships (28), or working in the sex industry (29, 30), all areas that carry additional health and psychological risks. Though the association between mental health and economic security may be stronger than physical health and economic insecurity (31), worry and distress about financial factors have been shown to be associated with worse overall physical health (31, 32). It is relevant to note that the evidence for the association between health and economic security relies on a variety of different economic stressors (e.g. housing, debt, employment, etc.), between studies, not allowing for a comparison of specific sources of economic stress.

Despite increased interest and an increased focus on healthcare and healthcare-related outcomes, there is no overarching framework to conceptualize the contributors to and components of economic (in)security at an individual level given multiple and varied conceptualizations of this concept. The lack of a common framework for economic security limits the field's ability to compare findings, synthesize lessons, and design interventions that address the root causes of threats to economic security. For health systems striving to address economic drivers of poor health, the lack of a shared language becomes a practical challenge: What, precisely, are we trying to change through intervention? What outcomes matter, and how should they be tracked over time?

The current study seeks to conduct a scoping review to uncover a framework for conceptualizing economic security by reviewing the literature and categorizing domains used to assess economic security. Such a framework can enable researchers, evaluators, and practitioners to align efforts, improve intervention design, and interrogate pathways through which economic conditions influence health. By doing so, we create the foundation for translational work that is not only rigorous but responsive to the realities facing patients and communities.

## 2 Methods

### 2.1 Study design

A scoping review of the literature was conducted to map how economic insecurity has been conceptualized and operationalized in relation to health and health-related functioning outcomes (33–35). We selected a scoping review methodology because our objective was conceptual clarification and structured synthesis rather than effect-size estimation or intervention evaluation (34, 35). Specifically, we aimed to (i) examine how economic insecurity has been defined, (ii) identify how it has been operationalized and measured, (iii) clarify how it has been linked to health and health-related functioning, and (iv) organize these elements into a conceptual framework reflecting patterns observed across studies.

Given substantial disciplinary variation in terminology and measurement approaches—spanning economics, public health, sociology, and health services research—a scoping review was appropriate for systematically mapping definitional and operational heterogeneity without restricting inclusion by study design. We completed the five core stages of Arksey and O'Malley's (33) scoping review framework (identifying the research question, identifying relevant literature, selecting studies, charting the data, and collating, synthesizing, and reporting the findings) while incorporating applicable methodological refinements described by Levac et al. (36). The optional stakeholder-consultation exercise described by Arksey and O'Malley was not conducted. Interdisciplinary review within the research team was incorporated into the analytic synthesis, as described below. Reporting aligns with PRISMA-ScR (37). Consistent with scoping methodology, we did not conduct a risk-of-bias assessment, as our purpose was to organize conceptual and measurement patterns rather than evaluate effectiveness (34, 35). A review protocol was developed for internal use but was not registered or made publicly available.

#### 2.1.1 Stage 1: defining the research question

Consistent with scoping review methodology, we articulated a research question broad enough to capture conceptual variation while structured to guide systematic mapping (36). Our overarching question was: “How has economic insecurity been conceptualized and operationalized in relation to health and health-related functioning outcomes in the peer-reviewed literature?” This framing emphasizes mapping how the construct is defined, measured, and positioned in health research rather than evaluating causal effects.

To structure the mapping process, we specified two guiding sub-questions: (1) How is economic insecurity defined and conceptually framed in studies examining health and health-related functioning?; (2) How is economic insecurity operationalized and measured?; These questions informed the development of the search strategy, eligibility criteria, data charting template, and analytic procedures (33, 36), and were designed to support systematic organization of existing conceptual and measurement approaches.

#### 2.1.2 Stage 2: identifying relevant literature

Guided by the research questions, we developed a comprehensive and iterative search strategy to capture variation in terminology describing economic insecurity in relation to health and health-related functioning outcomes (35, 36).

##### 2.1.2.1 Databases and timeframe

We searched PubMed and PsycINFO on July 14–15, 2025, thus coverage for the year 2025 is partial up to our search date. PubMed was selected to capture biomedical and health research literature, while PsycINFO ensured coverage of psychological and behavioral research, where economic insecurity is often conceptualized as a subjective experience. The search strategy combined economic security or insecurity with terms relevant to our goal of investigating operational definitions of this concept in the health literature. The final search combined the economic-security phrases with health- and measurement-related terms using the following Boolean structure: (“economic insecurity” OR “economic security”) AND (“health” OR “measure” OR “review”). We limited the search to articles published within the preceding 10 years (2015–2025) to reflect contemporary conceptualizations shaped by evolving labor markets and policy contexts (34).

##### 2.1.2.2 Scope and search yield

We restricted inclusion to peer-reviewed journal articles to support reproducibility and systematic comparison of conceptual and measurement approaches. Gray literature and non-peer-reviewed sources were excluded. The database searches returned 540 records from PubMed and 334 records from PsycINFO. Reference lists of articles included were manually reviewed to identify foundational contributions outside this timeframe; seven additional articles were identified through this process, thus a final yield of 881 records prior to duplicate removal. Screening procedures are described below.

#### 2.1.3 Stage 3: study selection

Following identification of potentially relevant articles, we applied predefined eligibility criteria to ensure that included studies contributed directly to conceptual or operational understanding of economic insecurity in relation to health and health-related functioning outcomes.

##### 2.1.3.1 Eligibility criteria

Studies were eligible if they (i) defined, conceptualized, measured, or reviewed economic insecurity or closely related constructs (e.g., economic security, financial hardship, precarity), and/or (ii) examined economic insecurity in relation to health or health-related functioning outcomes, including mental health, physical health, healthcare access, healthcare utilization, or functional wellbeing. To inform conceptual mapping, studies were required to provide either an explicit definition of economic insecurity or a clearly described operationalization. Articles referencing the term without definitional or measurement clarity were excluded. We excluded studies focused solely on macroeconomic indicators without a conceptual or empirical connection to individual- or household-level economic insecurity. We retained studies using area-level indicators when the authors explicitly presented those indicators as contextual measures or proxies of economic insecurity relevant to individual, household, or health-related outcomes. We also excluded non-peer-reviewed sources, and non-English publications. No restrictions were placed on study design; quantitative, qualitative, mixed-methods, conceptual, and review articles were all eligible. Consistent with scoping review methodology, we did not conduct methodological quality assessment because the objective was to organize conceptual and measurement patterns rather than evaluate study effectiveness.

##### 2.1.3.2 Screening procedures

A PRISMA flow diagram (Figure 1) summarizes the selection process (37, 38). After removal of 271 duplicates from 881 identified records, titles and abstracts were screened for eligibility. Seventy-two articles were advanced to full-text review. Nineteen were excluded during full-text review for insufficient definitional or operational detail, resulting in a final sample of 53 studies. Screening was conducted by two doctoral-level researchers experienced in scoping review methods (36). Screening decisions were made through a consensus-building, discussion-based process rather than independent dual screening. Thus, individual reviewer-level decisions, and formal inter-rater agreements statistics, were not generated. To account for this, a third doctoral-level researcher independently audited a random sample of 20 articles (10 included and 10 excluded), with 100% agreement.

**Figure 1:**
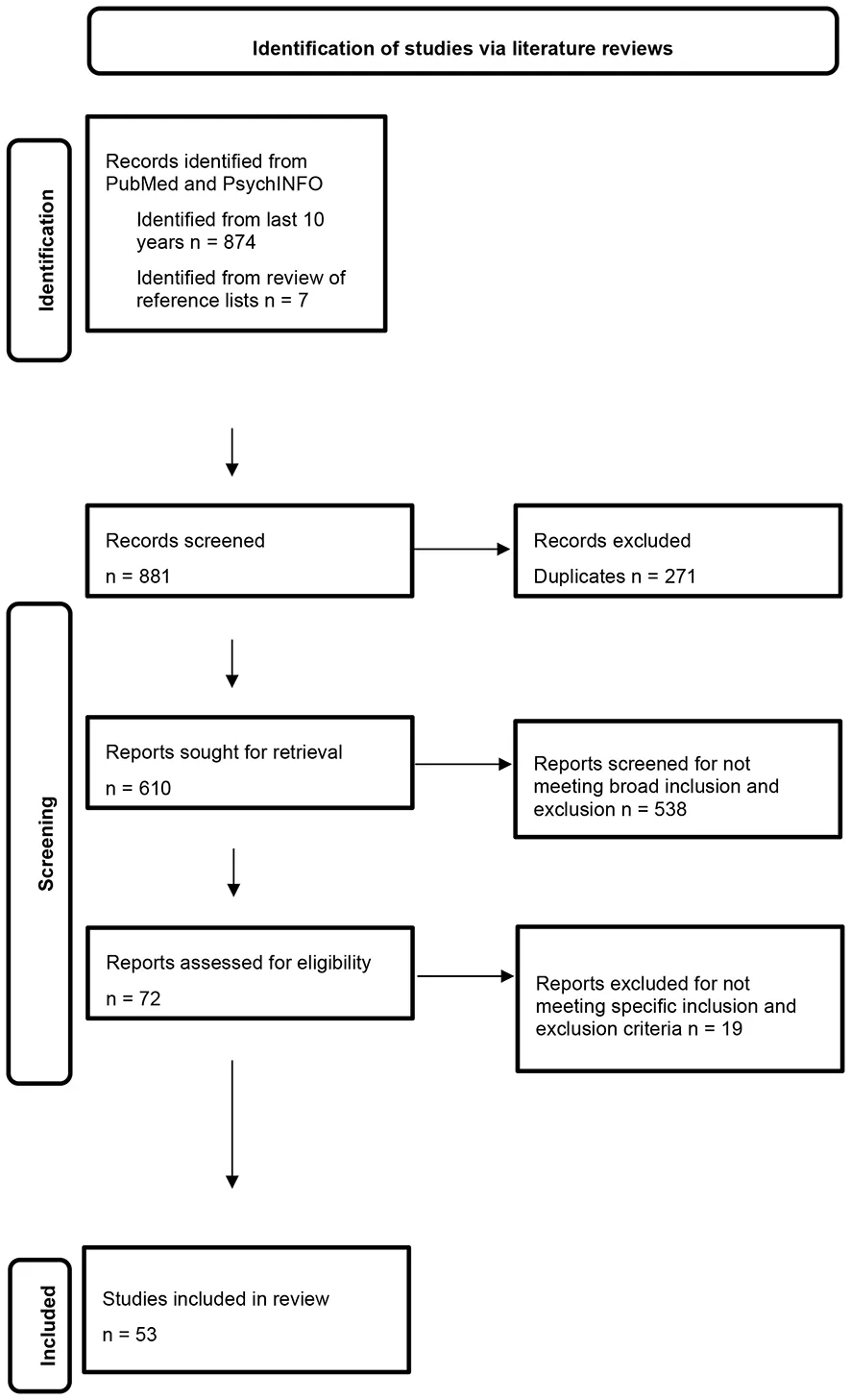
PRISMA chart.

#### 2.1.4 Stage 4: data charting

Following study selection, we conducted structured data extraction to systematically document information relevant to the guiding research questions and prepare for thematic synthesis (33, 36).

##### 2.1.4.1 Development of the charting approach

A standardized data charting template was developed by two doctoral-level researchers based on the research questions and pilot review of included studies (35). The template captured: study characteristics (publication year, country, article type), terminology used to describe economic insecurity, verbatim definitions (when provided), theoretical or conceptual framing, operational indicators and measurement strategies. The template intentionally distinguished between definitional content (how economic insecurity is conceptually described) and operational content (how it is measured). This separation was central to enabling later examination of alignment or divergence between conceptual framing and measurement practice. Minor refinements were made following pilot testing to clarify domain definitions and ensure consistent interpretation (36).

##### 2.1.4.2 Charting process

Each included article underwent full-text review and structured charting using the standardized template. Articles were independently reviewed by at least two team members. Verbatim definitions were recorded when available to preserve conceptual nuance. Operational indicators were extracted in sufficient detail to allow grouping into thematic domains during analysis; when composite measures were used, individual components were documented when described. Funding sources for the included studies were not charted. Regular team meetings were held to reconcile discrepancies and clarify domain boundaries. Differences in interpretation were resolved through consensus discussion. Study authors were not contacted to obtain or confirm charted information.

#### 2.1.5 Stage 5: analysis and synthesis

Following data charting, we conducted an iterative analytic process to organize definitional, operational, and relational elements identified across studies (35, 36). Consistent with scoping review methodology, the purpose of analysis was to identify patterns and structure conceptual variation rather than test hypotheses or evaluate causal relationships. Analysis proceeded in three phases – thematic analysis of definitions, thematic analysis of operational dimensions, and integrative synthesis to organize these elements into a conceptual framework.

##### 2.1.5.1 Thematic analysis of definitions

Verbatim definitions and relevant framing excerpts were analyzed using an inductive thematic approach (39). Initial coding identified recurring conceptual emphases, which were grouped into higher-order domains. We examined whether definitions emphasized subjective dimensions (e.g., perceived uncertainty, financial anxiety), objective dimensions (e.g., income instability, employment volatility, housing hardship), or hybrid formulations incorporating both. We also coded for cross-cutting characteristics, including temporal orientation (present hardship vs. anticipated future risk), risk framing, resource framing (stability versus adequacy), and unit of analysis (individual, household, structural). Coding was conducted independently by two researchers and refined through consensus discussion until definitional domains were stable (36).

##### 2.1.5.2 Thematic analysis of operational dimensions

Operational indicators were analyzed separately from definitions to avoid conflating conceptual framing with measurement practice. Measurement strategies were grouped into thematic domains such as employment instability, income volatility, housing hardship, insurance coverage, debt burden, and public assistance utilization. Sub-dimensions captured variation in measurement approach (e.g., objective versus perceived insecurity). We examined alignment between definitional framing and operationalization within each study to identify areas of conceptual-operational consistency or divergence. Coding was conducted independently and reconciled through discussion.

##### 2.1.5.3 Integrative synthesis and framework organization

Following separate analyses, we conducted an integrative synthesis to organize recurring patterns across studies. Through iterative comparison and diagrammatic mapping, we aligned core definitional domains, major operational categories, mechanisms described as linking economic insecurity to health, and contextual factors identified as shaping these relationships (36, 39).

##### 2.1.5.4 Interdisciplinary analytic review and refinement

Following development of the preliminary synthesis, members of the interdisciplinary research team reviewed the organization and interpretation of the identified domains, subdomains, and cross-cutting dimensions. This review drew on team expertise in health services research, social determinants of health, health economics, and implementation science and focused on the clarity of domain boundaries, consistency of terminology, distinctions between subjective and objective dimensions, and the representation of present conditions and future risk. Feedback from these discussions clarified terminology and domain boundaries and strengthened the organization of the subdomains without adding or removing any of the six overarching domains. No new empirical data or additional literature were introduced through this process. This interdisciplinary review was conducted as part of the research team's analytic synthesis and did not constitute formal consultation with external stakeholders or knowledge users.

## 3 Results

Fifty-three papers were included in the review (see Table 1). Forty-three papers were empirical studies, six were conceptual papers (meaning they did not attempt to answer a research question, but rather put forth a proposed definition of economic security), and four were literature reviews. Most of the studies (*n* = 29, 54.7%) were internationally focused, or non-US-based. Most papers (*n* = 42, 79.2%) referenced “economic insecurity” when providing definitions or construct operationalizations. Several other terms were used less frequently, including: “economic security,” “financial hardship,” “financial security,” “economic hardship,” and “precarity.” Analysis revealed both similarities and heterogeneities across the included studies. While most studies acknowledged multiple dimensions of economic security, the operationalization of these dimensions varied considerably in scope and emphasis.

**Table 1:** Overview of data extracted from articles reviewed.

References	Article type	Location	Term (s) Used	Definition	Dimensions of economic security
Abdul-Ghani et al. (68)	Empirical	United States	Economic insecurity	NR	1. Food insecurity (worried about running out of food), 2. Housing instability (sense of stability), 3. Financial concerns (perceived financial problems)
Abeyta et al. (57)	Empirical	United States	Financial security	“…an individual's sense that they can afford the things they need now and in the foreseeable futures.” (p. 692)	1. Perceptions of financial security (feeling secure, worrying about money, difficulty paying bills, concerns about financial future, difficulty affording necessities) 2. Current income level
Barakat and Konstandtinidis (7)	Review	N/A	Economic security	NR	1.Income amount and income sources 2. Times of hunger 3. Delivery (birth) payment mode 4. Deprivation 5. Having financial problems paying bills
Bjorklund (9)	Empirical	United States	Economic insecurity; precarity	“…income fluctuations resulting from involuntary wage changes (such as unstable employment or inconsistent pay) or unexpected income “shocks” (like sudden job loss or medical emergencies).” (p. 2)	1. Unemployment rate 2. Population percent with income below poverty level 3. Percent near FPL at 100% to 130% FPL 4. Rent burdened by spending 30% or more of income 5. Population percent under age 65 without health insurance 6. Percent of households receiving SNAP 7. Percent of households receiving SSI
Bossert and D'Ambrosio (46)	Conceptual	Canada	Economic insecurity	“…the anxiety produced by the possible exposure to adverse events and by the anticipation of the difficulty to recover from them. “ (p. 2)	1. Past and current wealth
Breiding et al. (27)	Empirical	United States	Economic insecurity	NR	1. Food insecurity (stress/worry about buying nutritious meals) 2. Housing insecurity (stress/worry about paying housing bill)
Choi et al. (69)	Empirical	United States	(Perceived) economic insecurity	NR	1. Difficulty paying bills, 2. Difficulty paying medical bills 3. Income loss
Chou et al. (58)	Empirical	United States	Economic insecurity	“…state of uncertainty and unpredictability regarding one's financial well-being; economic insecurity can manifest itself both concretely (e.g., as unemployment) and abstractly (e.g., as the possibility of future layoffs). That is, people derive their sense of economic insecurity from both their current economic situation and their perception of their future economic well-being.” (p. 444)	1. Household residents' employment status 2. State-level unemployment rate and individual employment status 3. Felt financially insecure, held an unstable job or worry about paying their bills 4. State-level unemployment 5. Poor job-related outcomes of recent graduates
Cochran et al. (70)	Empirical	United States	Economic hardship	NR	1. Food insecurity 2. Other general money problems (past week)
Conrad-Hiebner and Byram (53)	Review	N/A	Economic insecurity	“…a family's material hardship—food, bill paying, medical, and housing—employment status, debt, income, and income transfers from public or private sources. While public income transfers include government-funded social assistance programs (e.g., Supplemental Nutrition Assistance Program), private income transfers include private health insurance and financial assistance from friends or family.” (p. 158)	1. Earnings, income, cash assistance, poverty, poor, debt 2. Insurance/Medicaid 3. Temporary Assistance to Needy Families, Supplemental Nutrition Assistance Program, food stamps, 4. Earned Income Tax Credit, material hardship, economic hardship, economic insecurity, material deprivation
Fedina et al. (71)	Empirical	United States	Economic insecurity	NR	1.Access to food 2. Housing (cannot afford housing bill) 3. Health care (fill prescriptions/seek medical help) 4. Access to phone/internet services
Fedina et al. (72)	Empirical	United States	Economic insecurity	NR	1. Food insecurity (worry/stress about paying for food) 2. Housing insecurity (worry/stress about paying housing bill) 3. Healthcare insecurity (needed a doctor but couldn't afford it)
Goodman and Schneider (73)	Empirical	United States	Economic security	NR	1. Difficulty covering expenses and paying bills 2. Raise $400 in response to an unexpected need within the next month 3. Did not pay the full gas, oil, or electric bill in the past month 4. Hunger hardship (receiving free food or meals because they did not have enough money or going hungry but not eating in the last month)
Hacker et al. (55)	Empirical	United States	Economic security	“…the degree to which individuals are protected against hardship causing economic losses” (p. S7)	1. Probability of large household income declines 2. Possibility of large medical-out-of-pocket (MOOP) spending shocks 3. Capacity of households to buffer economic events by spending down liquid financial wealth
Holman and Walker (56)	Empirical	Europe (England and Wales)	Socio-economic security	“…levels of command over material and other resources over time.” (p. 247)	1. Income sufficiency (experienced- not cutting back on food or utilities) 2. Income security (experienced; no income drops; no arrears with bills/creditors; no increase in debt) 3. Income security (perceived; thinks finances will improve) 4. Housing security (experienced; no arrears on housing; no house loss) 5. Employment security (experienced; no job loss) 6. Working conditions (experienced; working less than 48 h in full-time job)
Hsieh (62)	Empirical	Russia and China	Economic security	NR	1. Believes household income is enough to cover daily living expenses 2. Perceived household financial situation 3. Having enough money to meet their needs
Hu et al. (23)	Empirical	China	Economic insecurity	“refers to the sense of uncertainty and unpredictability generated by individuals related to their economic status [(57, 58, 86)], including fear of unemployment, feeling that the economic situation may get worse, etc. (47).” (p. 2)	1. Economic insecurity of parents 2. Economic insecurity of self
Inoue et al. (74)	Empirical	Japan	Economic insecurity	NR	1. Economic anxiety
Knapp et al. (10)	Empirical	United States	Economic insecurity	“…the likelihood of a downward trajectory of income, material resources, and socioeconomic status” (p. 2131)	1. Not in labor force 2. Unemployment 3. Income spent on rent 4. Employed in service positions 5. Subprime credit rating (FICO credit score < 660)
Kong et al. (59)	Empirical	Canada	Economic insecurity	“…fears about potential future economic risks such as unemployment or ill health” (p. 1)	Worry about having enough money to support family
Kopasker et al. (47)	Empirical	United Kingdom	Economic insecurity	“…the anxiety produced by the possible exposure to adverse economic events and by the anticipation of the difficulty to recover from them (46, p. 1018).” (p. 184).	1. Qualifying income loss 2. Household wealth 3. Expected financial situation 4. Satisfaction with job security
Lee et al. (75)	Empirical	United States	Economic insecurity	NR	1. Income poverty 2. Material hardship (ability to pay rent, consistency of utilities, residential stability and having medical insurance)
Lee et al. (24)	Empirical	United States	(Pandemic related) Economic Insecurity	NR	1. Financial worries 2. Food insecurity 3. Lack of material resources to meet children's needs
Luo et al. (41)	Empirical	China	Economic insecurity	“the perception of an unavoidable and significant economic downside risk (40)” (p. 2)	1. Probability of being laid off 2. Average income loss
Morales et al. (60)	Empirical	United States	Economic insecurity	“…an individual's or family's anticipation of being able to support their basic material needs.” (p. 1707)	1. Individual economic insecurity (pay bills, pay for things, basic needs shortage, afford school supplies), 2. Family economic insecurity (poor housing or not having enough food and basic needs)
Mroz et al. (50)	Empirical	United States	Economic insecurity	“…the real and/or perceived risk of financial losses” (p. 1)	1. Difficulty paying usual expenses in past week (food, rent/mortgage, car payments, medical expenses, student loans, etc.) 2. Food insufficiency 3. Housing insecurity (behind on rent/mortgage)
Mutchler et al. (76)	Empirical	United States	Economic security; Economic well-being	NR	1. Housing costs 2. Food costs 3. Transportation costs 4. Medical care costs 5. Miscellaneous expenses also included (i.e., clothing, household items) 6. Unadjusted income
Niedzwiedz et al. (77)	Empirical	United Kingdom	Economic security	NR	1. Financial strain (perceived financial difficulty) 2. Difficulty paying bills, property taxes, or housing
Osberg (17)	Conceptual	Canada	Economic insecurity	“…the anxiety produced by a lack of economic safety, i.e. by an inability to obtain protection against subjectively significant potential economic losses” (p. 23)	1. Unemployment risk 2. Old age risk 3. Health risk 4. Widowhood/divorce risk
Osberg (40)	Conceptual	OECD Countries	Economic insecurity	“perceive a significant downside economic risk—i.e. a hazard or danger—looming in their economic future, which they are unable to adequately insure against or avoid or ignore” (p. 5)	1. Security of costs due to illness 2. Security of income due to widowhood 3. Security of costs due to disability 4. Security of income due to old age 5. Security of income due to unemployment
Osberg and Sharpe (42)	Conceptual	International (United States, United Kingdom, Canada, Australia, Norway, and Sweden)	Economic security	“Osberg (17) has argued that economic insecurity is, in a general sense, ‘the anxiety produced by a lack of economic safety—i.e., by an inability to obtain protection against subjectively significant potential economic losses”' (p. 305)	1. Unemployment risk 2. Illness risk 3. Single parent poverty risk 4. Poverty in old age risk
Osberg and Sharpe (43)	Conceptual	International (Canada, Denmark, Germany, US)	Economic insecurity	“…the anxiety produced by a lack of economic safety – i.e. by an inability to obtain protection against subjectively significant potential economic losses (17:17)” (p.5)	1. Unemployment risk 2. Illness risk 3. Single parent poverty risk 4. Poverty in old age risk 5. Risk of widowhood
Otaki et al. (78)	Empirical	Japan	Economic security	NR	Perceived financial difficulty to make ends meet
Prieto (61)	Empirical	Chile	Economic insecurity	“…'the adverse wellbeing effect of (involuntary) exposure to uncertainty in enduring an uninsured financial shortfall” [(65), p. 303]”' (p. 826)	1. Risk of unexpected economic event (income drop) 2. Unprotected workers or non-workers in household 3. Over-indebtedness 4. Asset poverty
Ranci et al. (51)	Empirical	Europe (Denmark, Sweden, UK, France, Italy, Spain, Hungary, Poland)	Economic insecurity	“…a situation in which the financial sustainability of the household is temporary threatened.” (p. 541)	1.Exposure to temporary poverty as result of income volatility 2. Household financial strain 3. Household incapacity to meet financial obligations and resulting over-indebtedness
Rebuffet et al. (79)	Empirical	France	Socio-economic insecurity; precariousness	NR	1. Have health insurance 2. Have support to help with difficulties 3. Seen family in past 6 months other than parents/children 4. Have someone to help financially 5. Live as a couple 6. Homeowner 7. Taken recent vacation 8. Participated in entertainment activities 9. Meet with social worker 10. Real financial difficulties (perceived) 10. Engaged in sport activities
Reeskens and Vandecasteele (80)	Empirical	Europe (24 countries)	Economic hardship	NR	1. Had to manage on a lower household income 2. Use savings or got into debt to cover living expenses 3. Cut back on holidays or new household equipment.
Richiardi and He (48)	Review	N/A	Economic insecurity	“…(i) the condition of not having “stable resources to support an adequate standard of living now and in the foreseeable future” (81), and (ii) the anxiety associated to it.” (p. 3)	N/A
Richiardi and He (49)	Conceptual	United Kingdom	Economic insecurity	“…the condition of not having access to stable resources to support an adequate standard of living now and in the foreseeable future (81).” (p. 2)	1. Forecast future income (relative to family size, age, and expenses) 2. Social welfare program availability
Roberts et al. (82)	Empirical	United States	Economic insecurity	NR	1. Current unemployment 2. Current lack of health insurance 3. Use of public assistance 4. Housing insecurity 5. Food insecurity
Rohde et al. (6)	Empirical	Australia	Economic insecurity	“…the anxiety felt by individuals when they are threatened by the prospect of severe economic losses” (p. 1)	1. Perceived job security 2. Financial satisfaction 3. Ease of getting emergency funds 4. Income level 5. Income drops
Rohde et al. (65)	Empirical	Australia	Economic insecurity	NR	1. Future income decline 2. Probability of future job loss 3. Subjective job security 4. Subjective difficulty raising emergency funds quickly
Rosal et al. (8)	Empirical	Puerto Rico	Economic insecurity	NR	1. Financial difficulty making ends meet
Sandoval-Heglund et al. (66)	Empirical	United States	Economic insecurity	NR	1. Food insecurity 2. Housing insecurity 3. Financial insecurity 4. Inability to afford medical care
Shin et al. (83)	Empirical	United States	Economic insecurity	NR	1. Current health insurance ownership 2. Household income 3. Current net worth 4. Employment status
Shuey and Willson (84)	Empirical	United States	Economic insecurity	NR	1. Poverty status 2. Asset poverty/wealth (above poverty threshold for 3 months) 3. Pension ownership 4. Home ownership
Staudigel (44)	Empirical	Russia	Economic insecurity	“…'significant downside economic risk—i.e. a hazard or danger—looming in their economic future, which they are unable to adequately insure against or avoid or ignore (40, p. 5).”' (p. 200)	1. Household income volatility 2. Probability of poverty 3. Unpaid bills 4. Wage arrears
Taylor et al. (85)	Empirical	United States	Financial hardship	NR	1.Unmet health care need due to cost 2. Health care unaffordability (worry about health care costs) 3. General financial stress (worry about bills, housing, credit cards, retirement, maintaining standard of living)
Tham et al. (67)	Empirical	United States	Economic security; Financial security	NR	1. Applied for/currently utilizing public benefits such as income assistance or food assistance or government health insurance
Watson and Osberg (26)	Empirical	Canada	Economic insecurity; economic security	Economic insecurity: “…the ‘inability to obtain protection against subjectively significant potential economic losses' (17, p.17)” (p. 119 )… “…a state of job-insecurity or joblessness” (p. 121). Economic security: “the state of being employed and having a perception of job security” (p. 120)	1. Perceived job security 2. Employment status (unemployed)
Western et al. (52)	Review	N/A	Economic insecurity	“…the risk of economic loss faced by workers and households as they encounter the unpredictable events of social life” (p. 342)	N/A
Yuan (45)	Empirical	Europe (28 countries and Israel)	Economic insecurity	“…anxiety about possible exposure to adverse economic events and the expectation of difficulty in recovery” (p. 2) and “…can be defined as ‘the inability to obtain protection against subjectively significant potential economic losses' (17).” (p. 4)	1. Postpone bill payments 2. Dipped into savings 3. Household's ability to make ends meet
Zahid et al. (54)	Empirical	United States	Economic insecurity	“…a multi-faceted concept that describesfinancial hardships due to effects of job loss, health crises, economic downturns, and insufficient safety nets” (p. 106)	2. Food insecurity 2. Financial wellbeing 3. Inability to pay emergency expense < $200 4. Housing insecurity

NR, not reported; N/A, not applicable.

### 3.1 Definitions of economic security

Twenty-eight articles provided explicit definitions of economic security. Analysis of these definitions revealed two primary cross-cutting dimensions: (i) subjective versus objective conceptualizations, and (ii) temporal orientation (present conditions versus future risk). The subjectivity/objectivity dimension was characterized as follows: subjective definitions are open to individual interpretations of experiences, whereas objective definitions are definitive responses and/or verifiable information that meet defined criteria. The temporal dimension was characterized as follows: an explicit or implicit reference to the time period by which economic insecurity is defined (i.e., past, present, or future).

Eight papers offered the same source definitions (17, 40) of economic insecurity (17, 26, 40–45). These papers described economic insecurity subjectively as an “anxiety” produced by a sense that one cannot protect themselves against future potential economic loss. Two additional papers (based on one definition) described economic insecurity as the subjective “anxiety” produced by possible exposure to future adverse events and anticipated difficulty recovering (46, 47). Two additional papers relied on a definition derived from the work of 48 (a monograph not reviewed in this study), which described economic insecurity subjectively as the “anxiety” associated with the condition of not having stable resources to support a standard of living in the present and in the future (48, 49). Across these twelve articles—drawn from three unique definitions—economic insecurity was defined as a subjective anxiety, and all refer to future economic prospects as contributing to current subjective anxiety. Only one article (26) offered distinct definitions of economic security and economic insecurity; however, operationally, these were measured as poles on a bipolar continuum on a measure of job security.

Sixteen additional papers offered unique definitions that were not quoted in another article. One paper defined economic security as both an objective and subjective state (50). However, most of these articles (*n* = 8), defined economic insecurity across varied objective terms based on the experience (or likelihood) of encountering economic shocks, risks, or threats (9, 10, 51, 52), having material or financial hardship (53, 54), having protection against hardship (55), or having command over resources (56). The remaining articles (*n* = 7) described economic insecurity as a negative subjective state such as uncertainty, anxiety, or fear (6, 23, 57–61).

References to temporal anchors (e.g., *past* vs. *current* vs. *future* financial hardship) were varied. Specifically, nine of the 16 articles that offered unique definitions of economic insecurity made implicit or explicit reference to *future* financial/economic risks (e.g., subjective worry or objective risk over future economic prospects) when defining economic insecurity (6, 10, 23, 56–61). The remaining articles lacked specific reference to future events, and thus appeared to center atemporal or ongoing concerns, such as “a family's material hardship” (9, 50–55).

### 3.2 Domains and sub-domains of economic security

We identified 51 articles that described domains and sub-domains of economic security, measuring or operationalizing its key components and/or indicators (Table 2). We uncovered six content areas that characterized these domains (ordered by referenced frequency): income/finances, healthcare, employment, housing, food, and wealth/assets. Most (*n* = 39) operationalized economic security across at least two domains. None included all six domains, and two included five domains.

**Table 2:** Analysis of domains and sub-domains used to operationalize economic security in articles reviewed.

Domains	Sub-domains	Articles cited
Income/Finances	*Financial hardship:* Inability to, or worrying about, paying bills/expenses (e.g., basic necessities, utilities, supplies for children/family, entertainment/leisure). Does not include housing, food, healthcare costs	Abeyta et al. (57), Barakat and Konstandtinidis (7), Choi et al. (69), Chou et al. (58), Cochran et al. (70), Fedina et al. (71), Goodman and Schneider (73), Holman and Walker (56), Hsieh (62), Hu et al. (23), Kong et al. (59), Lee et al. (75), Lee et al. (24), **Morales et al**. **(****60****)**, Mroz et al. (50), Niedzwiedz et al. (77), Otaki et al. (78), Ranci et al. (51), Rebuffet et al. (79), Reeskens and Vandecasteele (80), Rosal et al. (8), **Sandoval-Heglund et al**. **(****66****), Staudigel** **(****44****)**, Taylor et al. (85), Yuan (45)
	*Amount and source of income*: Individual or household income (sometimes used in reference to poverty threshold indicators or relative to expenses) and sources of income (e.g., using public benefits like SSI) presently and in the future	Abeyta et al. (57), Barakat and Konstandtinidis (7), Bjorklund (9), Conrad-Hiebner and Byram (53), Hacker et al. (55), Lee et al. (75), Mutchler et al. (76), Richiardi and He (49), Roberts et al. (82), Rohde et al. (6), Shin et al. (83), Shuey and Willson (84), Staudigel (44), Tham et al. (67)
	*Income losses*: Experiencing income drops or having less income than usual, or risk of income loss due to widowhood, divorce, or single parenting	Choi et al. (69), **Hacker et al**. **(****55****)**, Kopasker et al. (47), **Luo et al**. **(****41****), Osberg** **(****17****), Osberg** **(****40****)**, **Osberg and Sharpe** **(****42****)**, Osberg and Sharpe (43), Prieto (61), Ranci et al. (51), Reeskens and Vandecasteele (80), **Rohde et al**. **(****6****)**, **Staudigel** **(****44****)**
	*Perceived financial situation*: General subjective feelings about current financial status (e.g., feeling financially (un)stable, concerned about money, satisfaction with financial management, etc.)	Abdul-Ghani et al. (68), **Abeyta et al**. **(****57****)**, Chou et al. (58), **Holman and Walker** **(****56****)**, Hsieh (62), Inoue et al. (74), **Kopasker et al**. **(****47****)**, Niedzwiedz et al. (77), Rohde et al. (6), **Zahid et al**. **(****54****)**
	*Financial indicators*: Poor credit scores or over-indebtedness	Knapp et al. (10), Prieto (61), Ranci et al. (51)
	*Accessing savings*: Use of savings or emergency money for basic needs	Reeskens and Vandecasteele (80), Yuan (45)
Healthcare	*Healthcare hardship*: Worry about medical bills, or inability to pay healthcare costs	Barakat and Konstandtinidis (7), Choi et al. (69), Fedina et al. (71), Fedina et al. (72), **Sandoval-Heglund et al**. **(****66****), Taylor et al**. **(****85****)**
	*Healthcare vulnerability*: Potential risk to economic well-being due to financial costs associated with illness, disability, or old age	**Hacker et al**. **(****55****)**, Mutchler et al. (76), **Osberg** **(****17****), Osberg** **(****40****), Osberg and Sharpe** **(****42****), Osberg and Sharpe** **(****43****)**
	*Health insurance*: Having (or not) healthcare coverage	Bjorklund (9), Lee et al. (75), Rebuffet et al. (79), Roberts et al. (82), Shin et al. (83)
	*Healthcare assistance*: Utilizing public healthcare (e.g., Medicaid or Medicare)	Conrad-Hiebner and Byram (53), Lee et al. (75), Roberts et al. (82), Tham et al. (67)
Employment	*Employment status:* Individual, or household member, or community-level unemployment	Abeyta et al. (57), Bjorklund (9), Chou et al. (58), Knapp et al. (10), **Osberg** **(****17****), Osberg** **(****40****), Osberg and Sharpe** **(****42****), Osberg and Sharpe** **(****43****)**, Prieto (61), Roberts et al. (82), Shin et al. (83), Watson and Osberg (26)
	*Job stability:* Perceived job security or probability of job loss	Chou et al. (58), Kopasker et al. (47), **Luo et al**. **(****41****)**, Rohde et al. (6), **Rohde et al**. **(****65****)**, Watson and Osberg (26)
	*Employment quality:* Working under 48 hours per week, county percentage of service workers, unprotected workers in household	Chou et al. (58), Holman and Walker (56), Knapp et al. (10), Prieto (61)
Housing	*Housing hardship:* Inability to, or worrying about, paying housing bills	Breiding et al. (27), Choi et al. (69), Fedina et al. (71), Fedina et al. (72), Lee et al. (75), Niedzwiedz et al. (77), **Sandoval-Heglund et al**. **(****66****)**, Staudigel (44)
	*Perceived housing stability:* Subjective stability/security of housing	Abdul-Ghani et al. (68), Holman and Walker (56), **Morales et al**. **(****60****)**, Mroz et al. (50), Roberts et al. (82), **Zahid et al**. **(****54****)**
	*Homeownership:* Owning a home or paying a mortgage (vs. renting)	Fedina et al. (71), **Rebuffet et al**. **(****79****)**, Shuey and Willson (84)
	*Home burdened:* Rent or mortgage payments too high (relative to sources of income)	Bjorklund (9), Knapp et al. (10)
Food	*Food stability:* Having (or not having) enough nutritious food, or cutting back on food	Barakat and Konstandtinidis (7), Cochran et al. (70), Fedina et al. (71), Goodman and Schneider (73), Holman and Walker (56), Lee et al. (24), Mutchler et al. (76), Mroz et al. (50), Roberts et al. (82); **Sandoval-Heglund et al**. **(****66****)**
	*Food worry:* Subjective concerns related to getting enough food	Abdul-Ghani et al. (68), Breiding et al. (27), Fedina et al. (72), **Morales et al**. **(****60****)**, Roberts et al. (82), **Sandoval-Heglund et al**. **(****66****)**
	*Food assistance:* Utilizing public food assistance (e.g., SNAP), or receiving free food from other unspecified sources	Bjorklund (9), Conrad-Hiebner and Byram (53), Goodman and Schneider (73), Tham et al. (67)
Wealth/ Assets	*Amount of wealth/assets:* Individual or household asset totals (e.g., net worth, household wealth, asset poverty/material deprivation)	Barakat and Konstandtinidis (7), Bossert and D'Ambrosio (46), Conrad-Hiebner and Byram (53), Hacker et al. (55), Kopasker et al. (47), Prieto (61), Shin et al. (83), **Shuey and Willson** **(****84****)**
	*Savings worry:* Ability or concerns about having emergency funds to cover unexpected costs	**Goodman and Schneider** **(****73****)**, Rohde et al. (6); Rohde et al. (65), Zahid et al. (54)
	*Types of wealth/assets:* Source of individual or household assets (e.g., liquid cash, pensions, financial [or general] support from others)	Hacker et al. (55), Rebuffet et al. (79), Shuey and Willson (84)

Bolded citations use strategies for evaluating future risk to assess a given sub-domain of economic security.

The domains organize indicators according to their substantive content rather than their level of measurement. Although most indicators were measured at the individual or household level, several studies used county-, community-, or other area-level indicators as contextual proxies of economic insecurity. Inclusion within a shared domain does not imply that indicators measured at different levels are interchangeable.

Three to six sub-domains emerged within the six broad domains totaling 23 unique sub-domains. The top sub-domains included: financial hardship, amount or source of income, employment status, income loss, and perceived financial situation. Most (*n* = 17) included three sub-domains in their operationalization of economic security, though articles ranged from one to seven sub-domains. Descriptions of domains and sub-domains identified are provided below.

#### 3.2.1 Income/finances

The most referenced domain of economic security was individual and/or household income and general perceived financial difficulty. Most commonly (*n* = 25), researchers evaluated *financial hardship* as a sub-domain of economic security, which was characterized as difficulty paying bills or purchasing basic living needs other than housing, medical care, or food. This includes reports that an individual had unpaid bills, or that the individual was worried about their ability to pay their bills (mostly assessed in the present or past). Only four articles included assessments of future financial hardship. Many (*n* = 14) evaluated the *amount or source of income* in the past and/or present as indicators of economic security (i.e., determining if income falls below poverty level or relying on public assistance programs). Commonly (*n* = 13) researchers evaluated objective experience of *income loss* (e.g., experiencing drops in income or probability indices of population-level risk of future changes in income). Many (*n* = 10) assessed *perceived financial situation* to capture one's general sense of their financial life in the present and/or future. Less commonly, researchers evaluated other *financial indicators* (*n* = 3) such as population-level credit scores and personal over-indebtedness as well as *accessing savings* for everyday expenses (*n* = 2) as indicators of economic security. These were always present or past focused appraisals.

#### 3.2.2 Healthcare

Stable access to adequate and affordable healthcare was a frequently referenced domain of economic security, characterized by four sub-domains. Most commonly (*n* = 6) researchers evaluated current *healthcare hardship* to capture one's ability, or worry, to pay current or future medical expenses. Commonly (*n* = 6), studies evaluated *healthcare vulnerability* based on the extent that healthcare costs (present or future) pose a risk of economic hardship (e.g., large medical expenses, costs related to aging, illness, or disability). Some (*n* = 5) evaluated having or not having current *health insurance*. Less commonly (*n* = 4), individual and community-level *healthcare assistance* utilization, such as Medicare or Medicaid, were used as indicators of economic security.

#### 3.2.3 Employment

Another common domain of economic security was employment/unemployment. Most often (*n* = 12), researchers assessed present *employment status* to identify unemployment (at the individual or community-level). Multiple studies (*n*=6) described future economic risk as an individual's perceived *job stability* (or probability of job loss based on population-level data). *Employment quality* was evaluated across a few studies (*n* = 4), which captured hours worked per week, service-type work, and unprotected work as different indicators of economic security.

#### 3.2.4 Housing

Many researchers included housing as a domain when operationalizing economic security. Most commonly (*n* = 8), researchers evaluated *housing hardship* as an inability, or worry, to pay housing expenses. Additionally, researchers assessed *perceived housing stability* (*n* = 6), as an individual's perceived current and future security of their housing. Some evaluated *homeownership* (*n* = 3) or *home burdened* (*n* = 2)—housing costs that are too high relative to one's income—at the individual or community-level.

#### 3.2.5 Food

Many researchers evaluated food experiences when operationalizing economic security. Most commonly (*n* = 10), *food stability* was evaluated as an objective reduction or lack of nutritious food (i.e., “going hungry”). Some (*n* = 6) evaluated *food worry* related to purchasing food or the possibility of not having enough food. Others (*n* = 4) assessed receiving *food assistance* like SNAP or free food from other sources (at the individual or community-level).

#### 3.2.6 Wealth/assets

Wealth/assets were evaluated as an indicator of financial preparedness to manage economic shocks. Researchers often (*n* = 8) evaluated the *amount of wealth/assets* as a sub-domain of economic security. Less commonly, researchers evaluated *savings worry* (*n* = 4) and the *types of wealth/assets* (*n* = 3) such as liquid cash, pensions, or significant others who could lend financial support.

## 4 Discussion

This review advances the field by methodically mapping how economic insecurity has been defined, operationalized, and linked to health research, policy, and practice. Despite growing consensus that economic conditions shape health outcomes (3–5, 7, 52), our analysis reveals significant variability in how economic security is conceptualized, measured, and interpreted. Without clarity and alignment, researchers and health systems risk designing financial or psychosocial interventions that miss key mechanisms or fail to target the dimensions of economic security most consequential for health. To guide future operationalization and research, we present a conceptual framework of economic security as a visual synthesis of the dimensions identified in the literature based on topical domain, temporal considerations, and subjectivity (see Figure 2). This conceptual framework represents an organized synthesis of elements described in the reviewed literature. It does not introduce new theoretical constructs, specify causal directionality, or validate specific measures. Rather, it provides a structured representation of how economic insecurity has been defined, measured, and positioned in relation to health within existing research. Its purpose is to clarify conceptual boundaries, highlight areas of convergence and divergence, and support greater transparency in future measurement and study design. For purposes of this conceptual framework, economic security and economic insecurity are treated as opposite directions along a continuum, with greater insecurity reflecting poorer economic security. This organizing convention facilitates synthesis across studies using different terminology but does not presume that the continuum or its multidimensional structure has been empirically validated. As depicted in Figure 2, the reviewed literature characterized economic security across six interrelated domains (income/finances, healthcare, employment, housing, food, and wealth/assets) considered in relation to present reality and future risk. Figure 2 is intended only as a schematic organization of these reported dimensions; the overlap, proximity, and central placement of its elements do not represent empirically tested relationships, relative importance, or a validated underlying construct. Researchers further indicate that economic security may consist of both objective (e.g., wages) and subjective experiences (e.g., perceived financial hardship), likely with bi-directional impact. Each of these dimensions is explicated below.

**Figure 2:**
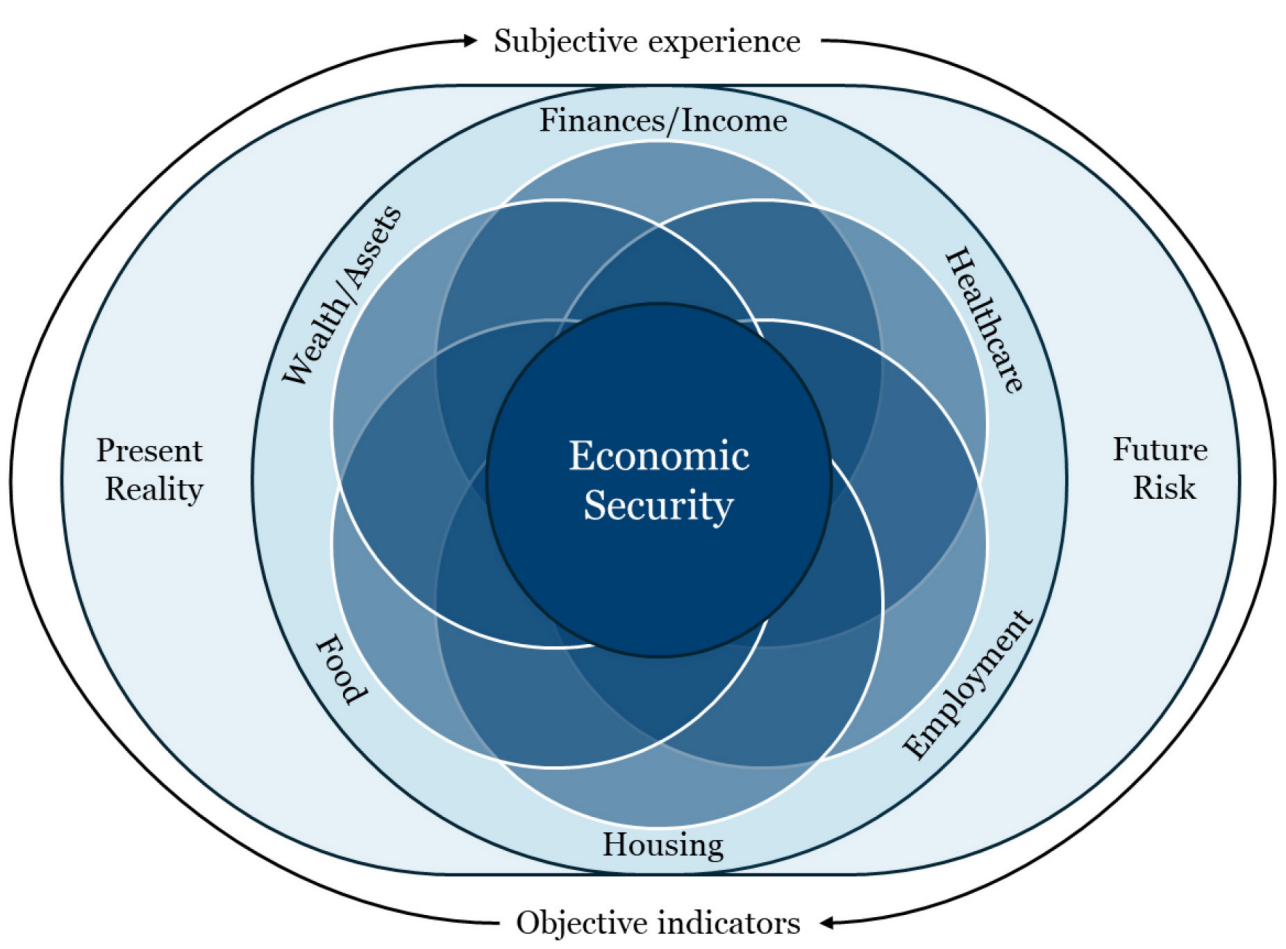
Visual framework of reported dimensions of economic security.

### 4.1 Domain considerations of economic security

This study reveals that researchers have converged on six domains of economic security: income/finances, healthcare, employment, housing, food, and wealth/assets. Across these domains, some seem to be capturing the current experiences as a proxy for economic security (i.e., cannot purchase food, cannot afford housing, lack of health insurance), others seem to be capturing antecedents of economic security (i.e., poverty wages, income losses, unemployment), while others are capturing future economic shocks (i.e., risk of job loss, risk of large medical expenses/illness). Future research should aim to clarify these distinctions to avoid conflating causes, consequences, and risk of economic security in ways that could limit interpretability or misdirect intervention design.

Our review also highlights a lack of uniformity in which domains and sub-domains of economic security are assessed. Most of the articles (76.5%) assessed at least two domains of economic security, and 86.3% assessed two or more sub-domains. There was a lack of consistency in which domains or sub-domains were evaluated, and why. The lack of a coherent conceptual model likely contributes to this variable approach to operationalizing economic security, which hampers empirical progress. For example, it is conceivable that many of these sub-domains are highly intercorrelated and may represent only one or two major domains. Disentangling the relationships within and between the domains and sub-domains would be helpful as they likely have varied impact on economic security and/or relationships among each other, which may differ in salience across populations or geographies such as immigration status, race, disability, gender, and other social positions. These identities/experiences likely intersect with economic security domains and sub-domains to shape distinct experiences of vulnerability. For example, it is plausible that housing instability may be a more immediate driver of economic security in urban areas, while income volatility or underemployment may be more salient in rural settings. Existing literature has documented differential associations between facets of economic security and health outcomes according to sociodemographic identities. For example, sex or gender-based findings were evident in terms of cognitive function (41), mental health (47), and body weight (44).

### 4.2 Levels of analysis and structural context

Consistent with the review's health-oriented research question and eligibility criteria, the included literature predominantly characterized economic security through individual- and household-level conditions and experiences. At the same time, several reviewed studies indicate that these experiences are shaped by broader contexts, including neighborhood conditions, national political and economic systems, and welfare-state arrangements (43, 51, 56, 62). Structural and institutional conditions may therefore influence the distribution, meaning, and health consequences of the domains identified in this review, but such conditions were not comprehensively represented in the reviewed literature. Accordingly, the framework is best understood as organizing the proximal domains through which economic security is experienced and measured, rather than as a comprehensive model of the upstream structural conditions that produce or modify economic security.

### 4.3 Temporal considerations of economic security

Our review highlights that the appraisal of one's current (or past) economic situation is a frequently used method to capture economic security, despite conceptual definitions emphasizing future risk. Ten sub-dimensions uncovered in this review included only past and/or present focused methods of operationalizing the respective sub-domain of economic security, which were typically focused on capturing objective indicators of present realities such as income level, or use of health or food assistance. Conversely, we found that economic security is most commonly defined in reference to future risk, such as subjective worry about potential financial losses. This disconnect in operationalizing economic security suggests a need to align measurement tools with conceptual definitions that capture how individuals experience and anticipate economic insecurities. The importance of temporal considerations in measurement has been reported by researchers comparing housing-related risk items. For example, De Marchis et al. (63) found differential housing-related risk and health consequences across subpopulations based, in part, on the temporal framing of questions (e.g., past, present, and/or future) with an indication that current and future-focused questions were related to worse health outcomes compared to past-focused questions.

### 4.4 Subjectivity/objectivity considerations of economic security

Researchers most commonly defined economic security as a subjective experience rather than objective indicators such as income or employment status. While subjective appraisals are essential for understanding how individuals internalize and respond to economic threats, they are not interchangeable with objective conditions (64). Moreover, research demonstrates that subjective evaluations and objective realities have differential impacts on outcomes. For example, subjective reports of economic security were related to perceived “meaning in life,” (57) worsened mental health (47, 65), and health-related patient outcomes (66) beyond objective measures such as income level.

Finally, though economic security and mental health outcomes can be related (6, 7, 26, 67), the frequent use of the term “anxiety” to subjectively define economic (in)security merits particular scrutiny. While this language underscores the psychological toll of precarious economic conditions, its application lacks specificity and may inadvertently pathologize normal responses (e.g., stress, worry) to material hardship. As health researchers, we must tread carefully to avoid conflating economic insecurity with anxiety without appropriate measures or diagnostic criteria.

### 4.5 Limitations

This review has several limitations. First, the search was limited to PubMed and PsycINFO, which were selected for their coverage of health, psychological, and behavioral research. Given the interdisciplinary nature of economic security, searching additional databases such as EconLit, Scopus, and Web of Science may have identified relevant work from economics, sociology, social welfare, and public policy. The selected databases, together with the exclusion of studies focused solely on macroeconomic indicators without a connection to individual- or household-level insecurity, may therefore have contributed to greater representation of health-oriented and proximal measures and less complete coverage of structural, institutional, and policy-level dimensions. Second, inclusion was limited to English-language, peer-reviewed publications, potentially excluding relevant perspectives from non-English and gray literature. Search terminology and the implementation of the eligibility criteria may also have resulted in missed studies. Although the recurrence of the identified domains across studies suggests that important conceptual patterns were captured, it does not establish that the framework is exhaustive. Third, we did not conduct formal consultation with external stakeholders or knowledge users. Although interdisciplinary review within the research team informed analytic refinement, consultation with people experiencing economic insecurity, practitioners, policymakers, and other knowledge users could have contributed additional perspectives on the framework's relevance, completeness, and applicability. Finally, the framework offers a guide for organizing existing conceptualizations and measures of economic security rather than a validated theoretical or causal model; it requires further refinement and testing before being used to explain, predict, or classify economic security.

### 4.6 Future directions

This paper offers a synthesis of the domains, temporal components, and subjective/objective aspects of economic security and presents an initial conceptual overview of these dimensions as a practical way to think about economic security, given current complexity and uncertainty about its definition. The conceptual overview synthesized here may guide intervention design, resource targeting, and the evaluation of structural reforms in health systems. For example, primary care teams incorporating routine screening to identify patients at risk for economic insecurity, or policymakers using multidimensional metrics to monitor economic security over time or across populations.

As a starting point, the conceptual framework requires further testing to determine which domains and dimensions are most pertinent to economic security across populations and contexts. Future research should also examine how the individual- and household-level domains synthesized here interact with upstream structural conditions, including labor and social protection policies, government social expenditures, inflation, regional economic conditions, and climate-related vulnerability. Linking individual- and household-level measures with policy, institutional, and area-level indicators through multilevel and longitudinal designs could clarify how structural conditions shape exposure to economic insecurity and modify its health consequences. In addition, future refinement should include consultation with people experiencing economic insecurity as well as with practitioners, policymakers, and other knowledge users to assess the framework's interpretability, relevance across settings and populations, and applicability to research, policy, and practice. Finally, reliable and valid measurement instruments are needed to assess the framework's dimensions, examine relationships within and across domains, predict relevant outcomes, and identify meaningful patterns of economic security.

## 5 Conclusion

Drawing from a variety of empirical, conceptual, and literature review papers, we sought to synthesize contemporary definitions and operationalizations of economic security. Our review highlights a lack of cohesion regarding defining and measuring economic security. To advance research and practice on economic security as a relevant factor in health, it may be helpful to standardize both conceptualization and measurement of economic security and its sub-domains. Our multidimensional conceptual framework serves as a starting point for such refinement of measurement approaches and empirical testing of the relationships between domains, with the goal of supporting more effective and scalable interventions to address economic drivers of health.

## Data Availability

The original contributions presented in the study are included in the article/Supplementary material, further inquiries can be directed to the corresponding author.
